# Chinese Home-Based Cardiac Rehabilitation Model Delivered by Smartphone Interaction Improves Clinical Outcomes in Patients With Coronary Heart Disease

**DOI:** 10.3389/fcvm.2021.731557

**Published:** 2021-10-05

**Authors:** Jing Ma, Cheng Ge, Yajun Shi, Yong Xu, Chenghui Zhao, Ling Gao, Dongling Wen, Tengjing Li, Jinli Wang, Sherry Yan, Sidney C. Smith, Yundai Chen

**Affiliations:** ^1^Department of Cardiology, First Medical Center of Chinese People's Liberation Army (PLA) General Hospital, Beijing, China; ^2^Center of Health System Research, Sutter Health, Walnut Creek, CA, United States; ^3^Heart and Vascular Center, University of North Carolina, Chapel Hill, NC, United States

**Keywords:** coronary heart disease (CHD), exercise training, smartphone, home-based cardiac rehabilitation, major adverse cardiac events

## Abstract

**Purpose:** We evaluated the long-term effect of a smartphone-facilitated home-based cardiac rehabilitation (HBCR) model in revascularized patients with coronary heart disease (CHD) on major adverse cardiac events (MACE), and secondary outcomes, including safety, quality of life, and physical capacity.

**Methods:** It was a prospective observational cohort study including a total of 335 CHD patients after successful percutaneous coronary intervention (PCI) referred to the CR clinic in China between July 23, 2015 and March 1, 2018. Patients were assigned to two groups: HBCR tailored by monitoring and telecommunication *via* smartphone app (WeChat) (HBCR group, *n* = 170) or usual care (control group, *n* = 165), with follow-up for up to 42 months. Propensity score matching was conducted to match patients in the HBCR group with those in the control group. The patients in the HBCR group received educational materials weekly and individualized exercise prescription monthly, and the control group only received 20-min education at baseline in the CR clinic. The primary outcome was MACE, analyzed by Cox regression models. The changes in the secondary outcomes were analyzed by paired *t*-test among the matched cohort.

**Results:** One hundred thirty-five HBCR patients were matched with the same number of control patients. Compared to the control group, the HBCR group had a much lower incidence of MACE (1.5 vs. 8.9%, *p* = 0.002), with adjusted HR = 0.21, 95% CI 0.07–0.85, and also had reduced unscheduled readmission (9.7 vs. 23.0%, *p* = 0.002), improved exercise capacity [maximal METs (6.2 vs. 5.1, *p* = 0.002)], higher Seattle Angina Questionnaire score, and better control of risk factors.

**Conclusions:** The Chinese HBCR model using smartphone interaction is a safe and effective approach to decrease cardiovascular risks of patients with CHD and improve patients' wellness.

**Clinical Trial Registration:** http://www.chictr.org.cn, identifier: ChiCTR1800015042.

## Introduction

Coronary heart disease (CHD) remains the leading cause of death worldwide after decades of major advances in treatment (1, 2). In the USA, over 370,000 people die annually from CHD (cdc.gov), whereas in economically developing countries, China, for instance, the incidence of CHD exceeds 11 million with a death rate of about 110 per 100,000.

Cardiac rehabilitation (CR), as a continuum of care for patients with CHD after initial treatment, has been approved to significantly promote wellness, improve exercise capacity, and preserve cardiac function (3, 4). In 2018, multiple medical professional agencies established referral to CR as a performance measure (5, 6). However, the referral rate and completion rate for CR are still suboptimal, with 53–74% of patients referred to CR (5, 6) and participation rates of 19–34% (7, 8). The participation rate is much lower in developing countries. In China, the referral rate is <1%, although a large number of patients are eligible (8). The major barriers to referral or participation to CR include lack of center-based cardiac rehabilitation (CBCR) facilities and lack of patient awareness. According to a survey, In China, there are only ~500 CR centers nationwide and mostly located in major cities; the majority of eligible patients are from rural areas and they do not have an access to CR. To increase participation and promote health and wellness, a more accessible and flexible model of CR is needed in China.

Home-based cardiac rehabilitation (HBCR) programs were thus introduced to increase access and patient acceptance and are reported to be equally effective as conventional CR in improving physical capacity and cholesterol control (9–12). HBCR, conducted at non-clinical settings, including home and other community-based facilities, is more accessible to patients and costing less (12). One study reported a more than 50% participant rate for HBCR in the United Kingdom after a cardiac event (13). However, compared to conventional CR, HBCR raised concerns about safety due to inadequate monitoring and instant communication with the care team. HBCR facilitated by telecommunication and monitoring offers a new opportunity.

HBCR with mobile communication has been used during the past decade, and studies, mostly conducted with text messaging, have shown promising results on improving CR enrollment, physical activity, and physical exercise capacity (14–17). The smartphone provides a flexible platform to deliver patient education, monitor physical activity, exchange patient data, and provide real-time communication and clinical support, and is a better form of telemedicine that can be delivered to patients compared to telephone call-/text message-based care (18, 19).

Most HBCR studies have revealed short-term benefits for CHD patients (i.e., 6 months or less) (14–16, 18, 20, 21), except one study with 24 months of follow-up, a reported exercise capacity, and clinical biometric outcomes (22). Major adverse cardiac events (MACE), however, are rarely reported in studies due to limited follow-up time. To this end, we evaluate the impact on MACE with up to 42 months follow-up, along with other clinical outcomes, including cardiovascular disease risk factors, exercise capacity, quality of life, and psychological outcome in a Chinese population. Similar to Dorje's study, we used WeChat, a social app widely used as the main tool to deliver HBCR with tele-education, telecommunication, telemonitoring, and data transferring functions (19).

## Methods

### Study Population

The study population included patients > 18 years of age who were referred to the cardiac rehabilitation clinic at First Medical Center of Chinese PLA General Hospital after successful PCI between July 23, 2015 and March 1, 2018. Successful PCI was defined as residual stenosis of the target lesion <30% without procedural complications and TIMI flow grade 3. Inclusion criteria also required smartphone ownership with an active WeChat account; 350 patients agreed to participate in the study.

After obtaining informed consent, all participants were rigorously screened for comorbidities, and 15 patients were excluded because they had any of the following conditions: unstable angina, myocardial infarction (MI) within 2 weeks, new ST-segment deviation, severe arrhythmias, decompensated heart failure, uncontrolled hypertension, severe pulmonary hypertension, obstructive hypertrophic cardiomyopathy, severe valvular heart disease, dementia, and inability to exercise as a result of orthopedic or neurological limitations. Among 335 remaining patients, 170 chose to participate in the smartphone HBCR program, while 165 patients declined and automatically became controls.

The study was approved by the ethics committee of Chinese PLA General Hospital and registered on http://www.chictr.org.cn (ChiCTR1800015042).

### Study Design and Procedures

This was a prospective observational cohort study. Participants were followed up to 42 months, and the last follow-up date was December 31, 2018. We stopped following up patients when the last follow-up date was reached, or patients had unscheduled rehospitalization due to worsening angina, or patients expired or developed a primary outcome. The primary outcome is the incidence of composite MACE, including cardiovascular death, non-fatal acute myocardial infarction, unscheduled coronary revascularization, and non-fatal stroke.

### Baseline Assessment for All Participants

Patients' demographics, socioeconomic status, disease history, current medications, and laboratory tests were collected at baseline, and physical examination was also conducted. Cardiopulmonary exercise testing (CPET) was performed to evaluate exercise intolerance and cardiopulmonary function (Supplementary Material).

A 12-lead electrocardiogram was monitored throughout CPET; the rated perceived exertion (RPE) on the original Borg scale was recorded at the end of each stage; oxygen uptake (VO_2_) and carbon dioxide output (VCO_2_) were measured every 10 s. Peak VO_2_ was defined as the average oxygen consumption during the last 15 s of cycle ergometry. VE/VCO_2_ slope was measured by plotting minute ventilation volume (VE) against VCO_2_ obtained every 10 s of exercise.

All cardiopulmonary exercise tests were reviewed by special medical staff blinded to the study protocol.

Patients completed baseline questionnaires to assess psychological stress, angina symptoms, and quality of life. Psychological stress was assessed by the Generalized Anxiety Disorder 7 (GAD-7) (23) and Patient Health Questionnaire 9 (PHQ-9) (24). The Seattle Angina Questionnaire (SAQ) was used to measure the effect of angina on physical limitation, anginal stability, anginal frequency, treatment satisfaction, and disease perception (25). The World Health Organization Quality of Life (WHOQOL) was used to assess the life quality of the participants.

All participants received 20 min of health education by CR doctors and nurses, including counseling on lifestyle modification, smoking cessation, and medication adherence.

All procedures complied with the *Helsinki Declaration* standards.

### CR Intervention Delivered by Smartphone

The CR intervention plan was based on standardized HBCR and secondary prevention guidelines (5, 26), including exercise prescription adjusted monthly and health education material.

#### Smart Phone Interaction System

The Smartphone Interaction System, a built-in WeChat plug-in app was developed by Halents Life-Info Technologies. It contains several modules, including the electronic medical management (EMM) software, an education module displaying educational materials; an exercise data collection module, which collects data from wearable devices; and a reminder module, to remind patients of upcoming clinic visits. The EMM software contains a database storing patient demographics, clinical measurement, and cardiopulmonary exercise testing results. An exercise prescription is also in the software, which sends prescriptions automatically to the intervention group monthly. After participant enrollment in the CR management system, remote data transmission makes the patient information accessible to both HBCR participants and staff.

Health educational materials were delivered to the intervention group weekly. The educational materials, including education about hypertension, diabetes, cardiovascular health, healthy nutritional advice, medications, psychological well-being, and smoking cessation, are in text-based education articles and video format. Educational topics also cover how to exercise and the appropriate exercise (e.g., exercise type, duration, intensity) for patients with CHD. The health educational materials were created based on evidence-based recommendations and were approved by a physician advisory board.

The heart rate, recorded by wearable devices such as monitoring watch or single chest straps, was monitored remotely by CR staff weekly. If the patient's heart rate exceeds the individualized target heart rate, the system will automatically alarm the participant *via* heart rate watch or smartphone and advise the patient to slow down. Besides uploading the heart rate records, a structured lab value collection table was sent to patients in this system; the patient can add body mass index (BMI) measure and lab values if new labs are available. Patients can also take a picture of lab reports and upload them through the system.

#### Exercise Prescription

An exercise prescription was determined using the target HR/HR_AT_ principle from the ninth edition of ACSM's guidelines for exercise (27). The target heart rate is defined as the sum of the resting heart rate or 50–80% of the reserve heart rate, sometimes combined with HR_AT_, depending on which value is lower than others. Exercise starts with 10 min of warm-up, followed by 30-min aerobic exercise (fast walking or cycling or slow jogging) or alternative exercise types to meet target heart rate, 10–15 minutes resistance, stretching,and balance training, and ended by 5-min cool down. Aerobic or stretching exercise is recommended five to six times weekly, while resistance and balance training are two to three times weekly. Patients were instructed to maintain all types of exercise intensity between “relatively easy” and “slightly tiring” which is equivalent to Borg Index 11–13 (28).

### MACE and Home Exercise Follow-Up

Clinic staff called or used WeChat to communicate with participants (HBCR and control groups) to collect MACE every 3 months. If an event occurred, the recall date and time were recorded. Patient medical records were also reviewed every 3 months by well-trained clinical staff to identify MACE events. Besides, several other questions were asked to patients, including whether exercise-related adverse events occurred, family support, length of exercise per day, and the number of days exercising per week. The length of exercise and number of days with exercise were scored according to the adherence scale developed by our group (29). Adherence to exercise is calculated based on a scoring algorithm: if a patient reported exercising 7 days a week was scored 5, 5–6 days scored 4, 3–4 days scored 3, 1–2 days scored 2, and not exercising scored 1. Patients exercising daily for more than 1 h scored 5, 30 min to 1 h scored 4, 10–30 min scored 3, and <10 min scored 2. For each individual, the total score is the product of the frequency score and duration of exercise score. A score above 12 (e.g., exercise 5–6 days a week and 10–30 min each time, or 3–4 days a week and 30 min−1 h each time) is considered good adherence to exercise.

An independent committee blinded to treatment assignment adjudicated clinical outcomes.

### Follow-Up Procedures

All participants (HBCR and control) were instructed to return to the clinic every 6 months for a follow-up visit. At the follow-up visit, blood pressure (BP), BMI, and waist-to-hip ratio were measured, and a series of tests were conducted, including lab tests [lipid panel, uric acid (UA), homocysteine (Hcy)], ECG, CPET, and echocardiogram. Patients were asked to answer GAD-7 and PHQ-9 questionnaires at follow-up visits. WHOQOL was administrated at the baseline and at the last follow-up. No incentives were given to participants who made follow-up visits.

### Control Group

Participants in the control group received standard care. Except for the same initial 20 min of education given to the intervention group at baseline, control participants did not receive any additional CR-related intervention.

### Statistical Analysis

Descriptive statistics were conducted for baseline measures, where continuous measures were summarized by the mean and standard deviation (SD), and categorical variables were summarized by proportion. To assess whether the HBCR group was similar to the control group at baseline measures, we conducted the Student *t*-test if the variables were normally distributed, or Wilcoxon rank-sum test otherwise for continuous variables, and the chi-square test for categorical variables.

We conducted propensity score matching (30), where propensity score was derived from a logistic regression model with HBCR/control status as the outcome, and with baseline characteristics (age, gender, BMI, systolic BP, physical capacity, comorbidities, and length of time between baseline and last follow-up time) to balance HBCR and control and to reduce the bias due to the natural limitation of the cohort study. Nearest neighbor matching within a 0.1 caliper distance was used to find matched cases and controls.

For the primary outcome (i.e., MACE event), a Kaplan–Meier survival plot was created and stratified by intervention group and control group, and the log-rank test was used to compare the survival probability. Multivariable Cox regression was used with (i.e., model 1 to model 3 in **Table 3**) and without (i.e. crude model in **Table 3**) adjustment for baseline characteristics. In the final model (model 3), baseline demographic, medication, and baseline exercise capacity were controlled. Hazard ratio and 95% confidence interval for intervention status were estimated.

Secondary outcomes included changes of CPET, symptoms, risk factors, exercise capacity, quality of life, and psychological outcomes. Changes in secondary outcomes were calculated by subcontracting baseline measures from follow-up measures. Paired *t*-test was used to compare changes between matched HBCR and control.

The absence of a measure for each secondary outcome during follow-up was assessed, if the overall absence rate was above 50% (in both HBCR and control groups), the outcome was dropped from the final analysis due to a high missing rate. For remaining secondary outcomes, we applied multiple imputations to impute missing values for follow-up measures; 100 imputed values for each missing measure were created based on Markov Chain Monte Carlo methods, and standard error was estimated based on Rubin's Rules wherein standard error was calculated by combining within-imputation variance and between-imputation variance (31).

In addition, as the comparison purpose, we conducted analyses for unmatched raw data as well as comparison. Similar multivariable Cox regression was applied to the primary outcome. For the secondary outcomes, due to potentially unbalanced baseline characteristics between HBCR and control groups, we applied the generalized linear model (GLM) to test the difference of changes between the HBCR group and control group after controlling baseline demographic and clinical variables. In order to adjust for different follow-up time, we also included time between the follow-up date and baseline date as a covariate in the model. The results were reported in Supplementary Material.

Statistical analysis was performed using SPSS 19.0 for Windows (SPSS Inc., Chicago, IL, USA) and SAS 9.2 (SAS Institute Inc., Cary, NC, USA).

## Results

At baseline, the average age was 56.3 (±9.5) years old, and the majority of participants were male (88%) and smokers (76%). Regarding comorbidities, 38% had an MI history, 55% had hypertension, 65% had hyperlipidemia, and 21% had diabetes. About half of the participants reached the targeted blood pressure (130/80 mmHg). Almost all patients received anti-platelet medications (98%), 81% statins, 59% β-blockers, and 22% ACEI/ARB (Table 1). The majority of participants in both the HBCR and control group did not report strong anxiety or depression. The average GAD-7 score was 3.24 (±4.14), and the PHQ-9 score was 4.46 (±3.7).

**Table 1:** Patients' baseline characteristics, by case and intervention groups.

	**Total (*n* = 335)**	**Control (*n* = 165)**	**HBCR (*n* = 170)**	***p*-value**
**Age (years)**	56.3 ± 9.5	56.5 ± 8.9	56.2 ± 10.1	0.755
**Sex, No**.
Male participant (%)	295 (88.1)	143 (86.7)	152 (89.4)	0.502
Female participant (%)	40 (11.9)	22 (13.3)	18 (10.6)	
Manual workers (%)	91 (27.2)	46 (27.9%)	45 (26.5%)	0.807
BMI [kg/m]	26.2 ± 3.0	26.2 ± 2.9	26.2 ± 3.1	0.896
WHR	0.93 ± 0.05	0.93 ± 0.05	0.93 ± 0.05	0.216
Exercise history (%)	184 (54.9)	82 (49.7%)	102 (60.0%)	0.063
Smoking history (%)	255 (76.1)	124 (75.2%)	131 (77.1%)	0.702
Systolic blood pressure (mmHg)	129.0 ± 14.1	128.7 ± 14.4	129.3 ± 13.9	0.699
Systolic blood pressure target-reached (%)	190 (56.7)	97 (58.8)	93 (54.7)	0.508
Diastolic blood pressure (mmHg)	81.0 ± 10.2	81.2 ± 10.3	80.9 ± 10.2	0.786
Diastolic blood pressure target-reached (%)	158 (47.2)	74 (44.8)	84 (49.4)	0.444
**Labs**
LDL (mmol/L)	1.97 ± 0.71	1.99 ± 0.75	1.95 ± 0.66	0.720
LDL target-reached (%)	85 (46.4)	40 (46.0)	45 (46.9)	1.000
Total cholesterol (mmol/L)	3.45 ± 0.88	3.48 ± 0.92	3.43 ± 0.85	0.670
Total triglyceride (mmol/L)	1.46 ± 0.90	1.51 ± 1.00	1.43 ± 0.81	0.581
Uric acid (μmol/L)	351.1 ± 80.0	347.8 ± 84.7	353.9 ± 76.1	0.625
Homocysteine (μmol/L)	15.4 ± 5.7	16.1 ± 6.5	14.9 ± 5.0	0.355
**Comorbidities**
Myocardial infarction history (%)	128 (38.2)	60 (36.4%)	68 (40.0%)	0.499
Hypertension (%)	184 (54.9)	87 (52.8%)	97 (57.1%)	0.444
Hyperlipidemia (%)	218 (65.1)	113 (68.5%)	105 (61.8%)	0.209
Diabetes mellitus (%)	70 (20.9)	28 (17.0%)	42 (24.7%)	0.106
**Echocardiography**
LVEF (%)	57.6 ± 8.9	59.0 ± 7.2	58.0 ± 7.1	0.182
Regional wall motion abnormality or ventricular aneurysm (%)	41 (12.2)	19 (11.5)	22 (12.9)	0.691
LVIDd (mm)	46.5 ± 4.7	46.4 ± 4.6	46.5 ± 4.8	0.861
IVS (mm)	10.8 ± 1.2	10.8 ± 1.1	10.8 ± 1.3	0.974
**Percutaneous coronary intervention**
Number of stents/patients	2.3 ± 1.4	2.2 ± 1.2	2.5 ± 1.6	0.071
Untreated stenosis (%)	187 (55.8)	87 (52.7%)	100 (58.8%)	0.273
**Medication**
Anti-platelet (%)	327 (97.6)	159 (96.4)	168 (98.8)	0.169
Statins (%)	271 (80.9)	133 (80.6)	138 (81.2)	0.502
β-Blocker (%)	198 (59.1)	95 (57.6)	104 (60.6)	0.581
ACEI/ARB (%)	74 (22.1)	34 (20.6)	40 (23.5)	0.513
Nitrates (%)	125 (37.3)	61 (37.0)	64 (37.6)	0.911
Diltiazem (%)	62 (18.5)	29 (17.6)	33 (19.4)	0.676
Trimetazidine (%)	119 (35.5)	57 (34.5)	62 (36.5)	0.733
**CPET**				
METS	5.43 ± 1.32	5.60 ± 1.31	5.27 ± 1.32	0.023^*^
Peak oxygen pulse (ml O_2_/beat)	11.70 ± 4.09	12.04 ± 3.64	11.38 ± 4.47	0.142
VO_2_ AT (ml.kg^−1^.min^−1^)	14.60 ± 4.43	15.08 ± 4.09	14.13 ± 4.71	0.050
VE/VCO_2_	25.50 ± 4.55	25.07 ± 4.81	25.92 ± 4.26	0.086
ΔVO_2_/ΔWR (ml.min^−1^.W^−1^)	11.72 ± 2.93	12.05 ± 3.13	11.39 ± 2.69	0.039^*^
**Psychological stress**
GAD-7	3.24 ± 4.14	3.62 ± 4.60	2.87 ± 3.63	0.107
PHQ-9	4.46 ± 3.70	4.78 ± 4.18	4.16 ± 3.16	0.137
**Symptoms**
SAQ-PL	67.01 ± 14.77	68.15 ± 13.95	66.08 ± 15.41	0.279
SAQ-AS	64.95 ± 29.40	64.53 ± 28.39	65.31 ± 30.31	0.837
SAQ-AF	88.81 ± 17.67	88.84 ± 18.77	88.78 ± 16.82	0.977
SAQ-TS	79.34 ± 14.10	78.44 ± 14.92	80.06 ± 13.41	0.377
SAQ-DP	60.51 ± 20.87	61.85 ± 22.25	59.43 ± 19.71	0.371

*BMI indicates body mass index; WHR, waist hip rate; LDL, low-density lipoprotein cholesterol; LVEF, left ventricular ejection fraction; LVIDd, left ventricular internal diameter at end-diastole; IVS, interventricular septum; ACEI, angiotensin converting enzyme inhibitor; ARB, adrenergic receptor blockers. Untreated Stenosis was defined as stenosis >50% left untreated after successful coronary stenting. METS indicates metabolism equivalents; VO_2_, oxygen consumption; AT, Anaerobic Threshold; VCO_2_, carbon dioxide production; VE/VCO_2_, minute ventilation/carbon dioxide production relationship; ΔVO_2_/ΔWR, VO_2_/work rate relationship; GAD-7, Generalized Anxiety Disorder-7; PHQ-9, Generalized Anxiety Disorder-7; SAQ, Seattle Angina Questionnaire; PL, physical limitation; AS, anginal stability; AF, anginal frequency; TS, treatment satisfaction; DP, disease perception. Continuous parameters are shown as mean ± standard deviation (SD). Categorical variables are presented as numbers and proportions. For continuous variables, comparisons between groups were made using two-sample t-test. Categorical variables were compared using the chi-square test*.

* *p value <0.05 was considered to be of statistical significance*.

The baseline characteristics of the HBCR group were similar to those of the control group, including demographics (age, gender), health behavior and lifestyle (smoking history, exercise history), vitals (BMI, BP), comorbidities (MI, hypertension, hyperlipidemia, diabetes), baseline lab measures (LDL, total cholesterol, uric acid, and homocysteine), echocardiographic results (LVEF, regional wall motion abnormality, LVID, and IVS), medication, PCI procedures, exercise capacity (heart rate reserve, peak oxygen pulse, VO_2_ AT, VE/VCO_2_), psychological stress, and symptoms (Table 1). Only two differences were observed for METS (5.3 ± 1.3 for HBCR group vs. 5.6 ± 1.3 for the control group, *p* = 0.02), and ΔVO_2_/ΔWR (12.1 ± 3.1 for control group vs. 11.4 ± 2.7 for the HBCR group, *p* = 0.04).

After matching, 135 patients remained in each group. The difference of baseline characteristics between HBCR and control groups were diminished, except for two lab measures (VO_2_ AT and ΔVO_2_/ΔWR) where measures for the control group were slightly higher than those in the HBCR group (*p* = 0.05 and 0.039, respectively, Supplementary Table 1).

There were no missing data for primary outcomes (Supplementary Table 2) and very little missing for MET, HR, and CPET measures at the final follow-up (0.9–1.2%). GAD-7 and PHQ-9 were missing for ~8% of participants. SAQ, LDL, TC, and TG have a moderate missing rate (35–49.9%), while UA and Hcy were not included in the final analysis due to the high missing rate (>50%). MET, HR, and CPET measures at 6, 12, 18, 24, or 30 months were also missing for more than half of participants; thus, these intermittent measures were not imputed and were not used in the analysis.

**Figure 1:**
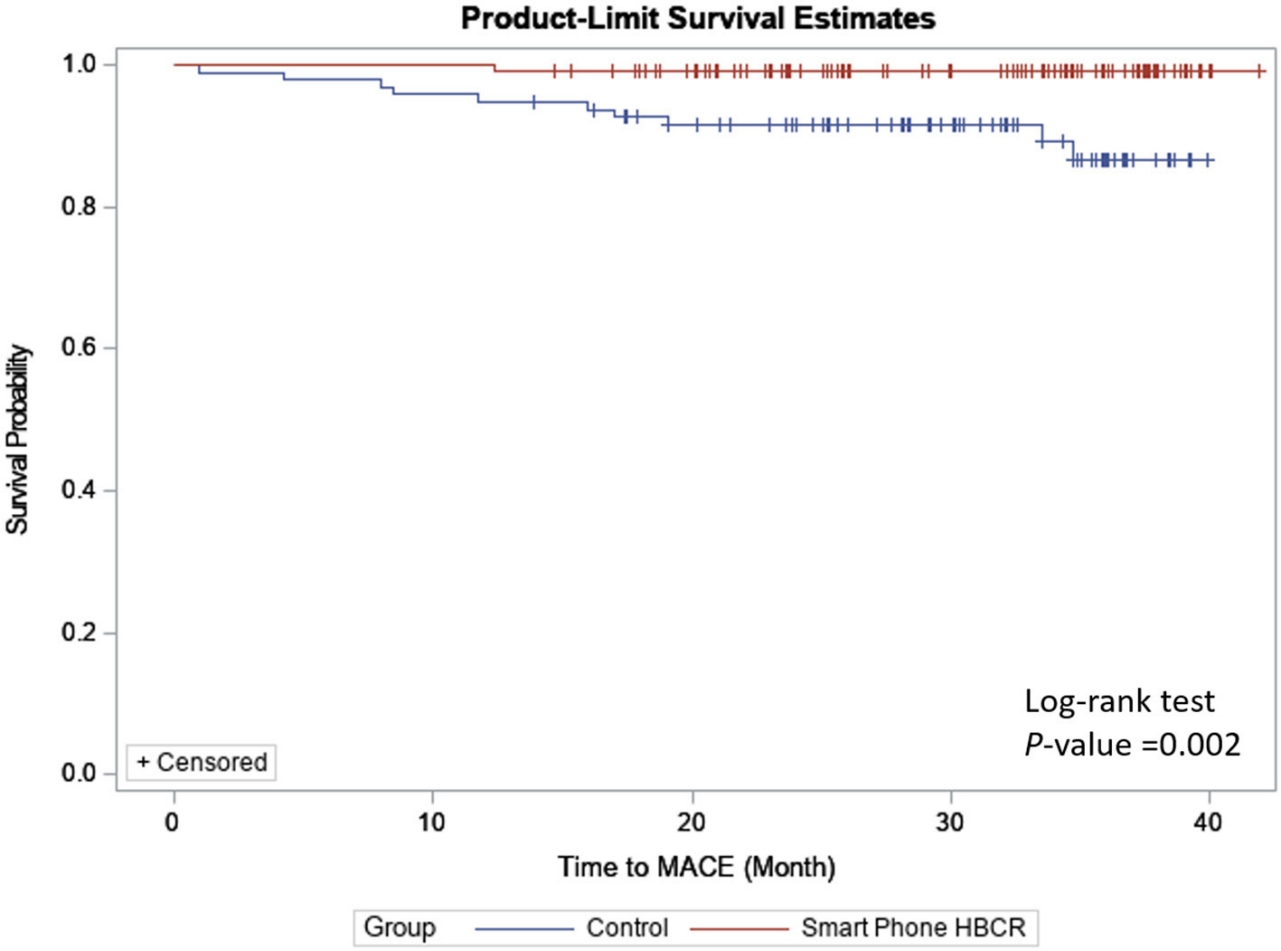
Kaplan–Meier plot for MACE, stratified by intervention status (for the matched cohort).

Among the matched cohort, a total of 14 (10.4%) MACE occurred during follow-up, including one cardiovascular death and 14 unscheduled revascularizations (Table 2). The HBCR group had a significantly lower incidence for MACE compared to the control group (1.5 vs. 8.9%, *p* = 0.002) (Figure 1); the hazard ratio of the incidence of MACE in HBCR reached 0.21 after adjusting for baseline characteristics (HR = 0.21, 95% CI 0.07–0.85) (Table 3, model 3).

**Table 2:** Summary statistics of the Home-Based Cardiac Rehabilitation program on primary and second outcomes at end of follow-up period for matched case and control.

		**Control(*n* = 135)**	**HBCR (*n* = 135)**	***p* ^†^**
**Primary outcomes**
**MACE**		12 (8.9)	2 (1.5)	0.002^*^
Incidence of myocardial infarction		0 (0.0)	0 (0.0)	–
Unscheduled revascularization		12 (8.9)	2 (1.5)	0.002^*^
Stroke		0 (0.0)	0 (0.0)	–
Cardiac death		1 (0.6)	0 (0.0)	0.493
**Second outcomes**
**Unscheduled hospitalization worsened due to worsening angina**		31 (23.0)	13 (9.7)	0.002^*^
**CPET**
METS		5.1 ± 1.4	6.2 ± 1.3	0.001^*^
Peak oxygen pulse (ml O_2_/beat)		12.4 ± 3.7	12.6 ± 3.4	0.27
VO_2_ AT (ml.kg^−1^.min^−1^)		13.7 ± 4.1	16.2 ± 4.3	<0.001^*^
VE/VCO_2_		25.4 ± 3.7	24.70 ± 3.8	0.34
ΔVO_2_/ΔWR (ml.min^−1^.W^−1^)		11.1 ± 2.9	11.9 ± 2.5	0.41
**Risk factors control**
SBP (mmHg)		130.1 ± 13.9	122.2 ± 13.7	<0.001^*^
Target-reached ratio of SBP		75 (55.5)	115 (85.2)	<0.001^*^
DBP (mmHg)		81.1 ± 10.4	77.5 ± 11.6	0.09
Target-reached ratio of DBP		76 (56.3)	99 (73.3)	0.03
LDL (mmol/L)		2.2 ± 0.8	1.5 ± 0.6	<0.001^*^
Target-reached ratio of LDL		55 (40.7)	118 (87.4)	<0.001^*^
**Other labs**
TC (mmol/L)		3.7 ± 1.2	3.1 ± 0.9	0.02^*^
TG (mmol/L)		1.6 ± 1.0	1.4 ± 0.9	0.12
UA (mmol/L)		340.0 ± 86.2	347.3 ± 77.9	0.71

*p^†^ is the p-value for raw data (without imputation)*.

*METS indicates metabolism equivalents; VO_2_ AT, oxygen consumption at Anaerobic Threshold; VE, pulmonary ventilation; VCO_2_, carbon dioxide production; VE/VCO_2_, minute ventilation/carbon dioxide production relationship; ΔVO_2_/ΔWR, VO_2_/work rate relationship; SBP, systolic blood pressure; DBP, diastolic blood pressure; LDL, low-density lipoprotein cholesterol; TC, total cholesterol; TG, total triglyceride; UA, uric acid*.

*Data are shown as mean ± standard deviation (SD)*.

*Time effects of exercise (±) on the second endpoints were analyzed using paired t-test*.

* *p-value <0.05 was considered to be of statistical significance*.

**Table 3:** Multivariate Cox regression analysis of the correlation between Home-Based Cardiac Rehabilitation program and incidence of clinical events, with and without adjusting for confounding variables.

	**HR (95% CI)**	***p*-value**
Crude	0.21 (0.07, 0.85)	0.008
Model 1	0.21 (0.070, 0.85)	0.008
Model 2	0.21 (0.07, 0.85)	0.008
Model 3^*^	0.21 (0.07, 0.85)^*^	0.008^*^

* *The fully adjusted result, which is used in interpreting the association between Home-Based Exercise and Incidence of Clinical events*.

*Model 1: after adjustment for age and gender*.

*Model 2: after further adjustment for history of previous myocardial infarction, history of diabetes mellitus, number of stents implanted, whether has residual stenosis or not*.

*Model 3: after further adjustment for BMI, WHR, history of Hypertension, history of smoking, history of exercise, maximal METs, oxygen uptake at AT, VE/VCO_2_, ΔVO_2_/ΔWR, use of at least one antiplatelet drug, use of statins, use of trimetazidine, use ofβ-blockade, Whether received emergency PCI or not, systolic blood pressure value and diastolic blood pressure value at the baseline level*.

*BMI indicates body mass index; WHR, waist hip rate; METS, metabolism equivalents; AT, anaerobic threshold; VE/VCO_2_, minute ventilation/carbon dioxide production relationship; ΔVO_2_/ΔWR, VO_2_/work rate relationship; PCI, percutaneous coronary intervention*.

The participants in the HBCR group demonstrated much lower incidence of unscheduled hospitalizations due to worsened angina than those in the control group (23.0 vs. 9.7%, *p* = 0.002). The exercise capacities in the HBCR group were also better at the end of follow-up compared to controls: maximal METs were higher in the HBCR group (6.2 vs. 5.1, *p* = 0.001) than that for the control group, and oxygen uptake at anaerobic threshold (VO_2_ AT) was also improved (16.2 vs. 13.7, *p* < 0.001) in the HBCR group. Regarding the risk factor control, at the final follow-up, the HBCR group had lower SBP (122 vs. 130, *p* < 0.001) and a higher proportion of patients with controlled systolic blood pressure (85.2 vs. 55.5%, *p* < 0.001) as well as diastolic blood pressure (73.3 vs. 56.3%, *p* = 0.03). Similarly, the HBCR group also had lower LDL-C (1.5 vs. 2.2, *p* < 0.001) and a higher proportion of patients with controlled LDL-C (87.4 vs. 40.7%, *p* < 0.001) (Table 2).

Table 4 shows the changes in secondary outcomes using imputed data. Among CPET measures, the HBCR group (mean = 0.9, 95% CI: 0.9–1.5) showed significant increase in METs compared to the control group (mean = 0, 95% CI: −0.3 to 0.1). The HBCR groups also had more significant increase in VO_2_ at AT (mean = 1.9, 95% CI 0.9–2.2), ΔVO_2_/ΔWR (mean = 0.6, 95% CI −0.1 to 0.9) and decrease in VE/VCO_2_ (mean −1.2, 95% CI −1.6 to 0.6) compared to controls. Systolic BP was reduced for the HBCR group (mean = −5.2, 95% CI = −7.7 to 1.1), but not for the control group (mean = 0.5, 94% CI −2.0 to 3.0). The differences of those changes between the HBCR and control group were significant (*p* = 0.003).

**Table 4:** Effects of the Home-Based Cardiac rehabilitation program on psychological state, cardiac symptoms and biochemical metric changes for the matched cohort.

	**Control Change^†^ (*n* = 135) Mean (95%CI)**	**HBCRChange^†^(*n* = 135)Mean (95%CI)**	**Difference (HBCR-Control) Mean (95% CI)**	***p* ^**††**^**
**Psychological stress**
GAD-7	−0.2 (−0.9, 0.4)	−0.5 (−1.0, 0.5)	−0.3 (−1.4, 7)	0.20
PHQ-9	0.2 (−0.4, 0.8)	−0.3 (−0.9, 0.5)	−0.5 (−1.3, 0.6)	0.22
**Symptoms**
SAQ-PL	−0.7 (−4.1, 2.2)	6.9 (3.8, 10.2)	7.6 (3.2, 12.1)	0.001
SAQ-AS	−1.9 (−10.1, 5.3)	5.9 (−1.4, 12.8)	7.7 (−1.9, 13.8)	0.09
SAQ-AF	2.0 (−0.4, 6.0)	6.9 (4.2, 10.7)	4.9 (1.0, 7.4)	0.04
SAQ-TS	0.8 (−2.1, 4.0)	2.9 (−0.4, 5.9)	2.1 (−1.7, 5.1)	0.21
SAQ-DP	4.3 (1.9, 10.2)	8.9 (4.1, 13.4)	4.6 (0.9, 6.1)	0.04
**Biometric metric**
SBP	0.5 (−2.0, 3.0)	−5.2 (−7.7, −1.1)	−5.7 (−7.8, −2.2)	0.003
DBP	−0.7 (−2.2, 0.7)	−3.2 (−4.4, −0.9)	−2.5 (−3.5, 0.2)	0.09
LDL-C	0.2 (−1.0, 1.3)	−0.3 (−1.3, 0.7)	−0.5 (−1.7, −0.1)	0.03
**CPET**
Maximal MET	0.0 (−0.3, 0.1)	0.9 (0.7, 1.2)	0.9 (0.9, 1.5)	<0.001
Peak oxygen pulse (ml O_2_/beat)	0.0 (−0.3, 0.6)	1.8 (0.7, 2.0)	1.8 (0.4, 1.7)	0.05
VO_2_ AT (ml.kg^−1^.min^−1^)	−0.7 (−1.4, −0.2)	1.9 (0.9, 2.2)	2.6 (1.6, 3.3)	<0.001
VE/VCO_2_	0.4 (−0.6, 1.2)	−1.2 (−1.6, −0.6)	−1.6 (−2.1, −0.7)	0.001
ΔVO_2_/ΔWR (ml.min^−1^.W^−1^)	−0.6 (−1.3, −0.1)	0.6 (−0.1, 0.9)	1.2 (0.8, 1.5)	0.004

† *Changes were summarized based on imputed data*.

*^††^p is the p-value after imputation to test the changes between the HBCR and control groups*.

*GAD-7 indicates Generalized Anxiety Disorder-7; PHQ-9, Generalized Anxiety Disorder-7; SAQ, Seattle Angina Questionnaire; PL, physical limitation; AS, anginal stability; AF, anginal frequency; TS, treatment satisfaction; DP, disease perception; SBP, systolic blood pressure; DBP, diastolic blood pressure; METS, metabolism equivalents; VO_2_, oxygen consumption; AT, anaerobic threshold; VCO_2_, carbon dioxide production; VE/VCO_2_, minute ventilation/carbon dioxide production relationship; ΔVO_2_/ΔWR, VO_2_/work rate relationship*.

*p-value <0.05 was considered to be of statistical significance.

We assessed the psychological status and cardiac symptoms at the final follow-up (Table 4). Among matched patients, the changes of GAD-7 and PHD-9 did not show significant difference between the HBCR and control groups (*p* > 0.20), while three dimensions of SAQ showed significant difference between the HBCR and the control groups, which were SAQ physical limitation, angina frequency, and disease perception. The SAQ physical limitation measure increased 6.9 (95% CI: 3.8–10.2) for the HBCR group, which was significantly higher than that for the control group (mean = −0.7, 95% CI: −4.1 to 2.2) (*p* = 0.001). A similar tendency was found in the increase of SAQ angina frequency and disease perception (2.9 vs. 0.8, *p* = 0.04).

## Discussion

This HBCR model which delivers via smartphone using the WeChat built-in application in China found a significant reduction of MACE in the HBCR group compared to the control group (1.5 vs. 8.9%), confirming a clear benefit for HBCR during the 24–48-month follow-up period. To our knowledge, this is the first published study to assess the effect of smartphone-based HBCR programs on MACE for patients with CHD with a long follow-up time. No physical exercise-related adverse events were observed during the follow-up period for both groups.

MACE is rarely reported as an outcome in CR studies. Soga et al. reported the impact of exercise training on MACE events after coronary stenting for CHD patients within 30 days as a safety concern of early exercise after the coronary procedure (32). In our study, we extend the safety concern of HBCR to a long-term effectiveness measure, and MACE was evaluated after more than 2 years of follow-up. Smartphone-facilitated HBCR can effectively reduce MACE and is suggested to be suitable for long-term use to prevent secondary severe outcomes in order to promote general wellness to post-cardiac patients.

Consistent with another HBCR study, our study indicated improved exercise capacity for patients, particularly for METs, and oxygen consumption at Anaerobic Threshold (VO_2_ AT) (Table 2). Moreover, our study showed that the improvement persists longer than 2 years (3, 9, 10). Previous studies have established the connection between exercise capacity and health outcome: each increment of 1 metabolic equivalent (MET) (3.5 ml O_2_ kg^−1^.min^−1^) in peak VO_2_ corresponds to 13% reductions in all-cause mortality and 15% in cardiovascular mortality (32). In our study, although the baseline levels of peak METs, oxygen uptake, and ΔVO_2_/ΔWR in the control group were slightly higher than those in the HBCR group, after 2 years of intervention, peak METS were significantly improved in the HBCR group. Moreover, VO_2_ at the anaerobic threshold (VO_2AT_) was observed to be higher in the HBCR group than the control group, which indicates the further benefit of HBCR on cardiorespiratory fitness. The improvements of exercise capacity in the HBCR group were also more significant than those in the control group.

Our study also found better adherence to exercise in the HBCR group (85%) compared to the control (31%) during the entire follow-up period, suggesting that smartphone-based HBCR can be used as a long-term secondary prevention tool. Though it is not clear which components of this smartphone-based HBCR directly lead to improved exercise adherence, the real-time monitoring, communication about exercise-related concerns, individualized exercise prescription, and repeated education may work together to promote patient engagement. Regardless of the mechanism, long-term adherence to exercise, resulting in improved exercise capacity, was associated with a reduction of MACE.

Improved LDL-C and systolic BP control observed at the final follow-up visit were consistent with findings from another smartphone-based HBCR study (19). In that study, systolic BP in HBCR was 8 mmHg lower compared to the control group at 6-month follow-up, which is slightly higher than the 5 mmHg reduction in our study after 2 years of follow-up. LDL-C was reduced 0.5 mmol/L more for HBCR in our study, compared to 0.22 mmol/L in Dorje's study at 12-month follow-up (19). It has been suggested that the 10 mmHg reduction of systolic BP is associated with 20% reduction of major cardiovascular events (e.g., stroke, MI, HF) (33, 34). Although our results did not reach the same degree of blood pressure reduction, the proportion of patients who achieved the target BP control was significantly improved (55% at baseline vs. 85% at the final follow-up visit) for the HBCR group, compared to worsened BP control for the control group (59% at baseline vs. 56% at the final follow-up visit) (Tables 1, 2). Similarly, we also observed significantly improved LDL-C control proportion (47 vs. 87%) for HBCR, compared to 46 vs. 41% for the control group (Tables 1, 2). BP and LDL-C control are closely related to lifestyle modification (e.g., exercise, diet), medication adherence, the educational material, exercise prescription, and monitoring provided to the HBCR group, which are likely to be associated with improved BP and LDL-C (27). Moreover, LDL-C control is directly associated with the reduced risks of cardiovascular disease and has been strongly recommended by clinical guidelines since 2000. Educational materials in the present study, particularly lifestyle modification and diet education (35, 36), are thought to contribute to the improvement in BP and LDL-C.

Smartphone-based HBCR makes it possible for real-time communication and data exchange between medical staff and patients (*via* WeChat) and promotes personalized exercise prescription and exercise safety. Besides this individualized care, which is usually an advantage of center-based CR, smartphone-based HBCR is conducted in the home environment and is more acceptable and accessible for patients. Thus, smartphone-based intervention represents a new form of telemedicine wherein care can be effectively delivered through telecommunication. This form of telemedicine is particularly useful when patients are not able to access clinic facilities (e.g., current lockdown due to COVID-19). It is also a better option for countries such as China where the smartphone is commonly available but health care facilities are limited, particularly if intervention focuses on improving knowledge and awareness of the disease through educational materials. Moreover, a social media communication tool such as WeChat has widespread use and extended functions that can be connected to other applications (e.g., wearable devices) thereby making the social media tool (e.g., WeChat) an ideal and flexible platform to deliver care (19). It can be easy to adapt as one form of telemedicine. The experience from our study should be easily generalized to other disease areas.

There are several limitations of the study. First, participants were not randomized and participant assignment was based on their willingness to participate in HBCR which may result in a self-selection bias. We conducted propensity score matching (30) to reduce the potential bias; the baseline characteristics were balanced among HBCR and control groups among the matched cohort. The impact of HBCR in reducing MACE was even more appealing compared to the unmatched cohort (Supplementary Tables).

Secondly, the sample size was relatively small and all patients were recruited from a single center. Therefore, a large-scale multi-center is needed to further verify the results. A multi-center smartphone-based HBCR randomized trial, led by our clinic center, has been initiated and is now in the patient recruitment stage. Nevertheless, randomized control trials with a greater number of patients are needed to confirm the findings of this study.

Thirdly, because all follow-up visits are voluntary, more than half of patients missed intermittent follow-up measures because of secondary outcomes. Patients have to pay a ~500 Chinese Yuan (~US $70) out-of-pocket expense at each follow-up visit, which is a financial burden for most patients. Ideally, health insurance would pay for tests as a continuum of care for CHD patients. Because it generally takes a long time to advocate health policy change, this may limit the practical use of HCBR.

## Conclusion

To our knowledge, this study provides the longest follow-up evaluation of smartphone-based HBCR for successfully revascularized CHD patients. After more than 2 years of follow-up, smartphone-based HBCR facilitated by a social network application (WeChat) was effective in decreasing the incidence of major adverse cardiovascular events, improving exercise capacity, and risk factor control.

## Data Availability Statement

The raw data supporting the conclusions of this article will be made available by the authors.

## Ethics Statement

The study involved human subjects and was approved by the Ethics Committee of the Chinese PLA general hospital. The participants provided written consent to the study.

## Author Contributions

The study was initiated by JM and YC. JM, CG, and SY performed the statistical analysis and drafted the manuscript. YS, YX, CZ, LG, DW, TL, and JW were helpful for data collection. YC and SS contributed substantially to its revision. All authors contributed to the article and approved the submitted version.

## Funding

This work was supported by the National Key R&D Program of China (2018YFC2000600).

## Conflict of Interest

The authors declare that the research was conducted in the absence of any commercial or financial relationships that could be construed as a potential conflict of interest.

## Publisher's Note

All claims expressed in this article are solely those of the authors and do not necessarily represent those of their affiliated organizations, or those of the publisher, the editors and the reviewers. Any product that may be evaluated in this article, or claim that may be made by its manufacturer, is not guaranteed or endorsed by the publisher.

## References

[1] Bloom DE Cafiero E Jané-Llopis E Abrahams-Gessel S Bloom LR Fathima S. The global economic burden of noncommunicable diseases. In: Pgda Working Papers (2012).

[2] Briffa TG Tonkin A. Put disease prevention first. Circulation. (2013) 128:573–75. 10.1161/CIRCULATIONAHA.113.004416 23836836

[3] Taylor RS Brown A Ebrahim S Jolliffe J Noorani H Rees K. Exercise-based rehabilitation for patients with coronary heart disease: systematic review and meta-analysis of randomized controlled trials. Am J Med. (2004) 116:682. 10.1016/j.amjmed.2004.01.009 15121495

[4] Goel K Lennon RJ Tilbury RT Squires RW Thomas RJ. Impact of cardiac rehabilitation on mortality and cardiovascular events after percutaneous coronary intervention in the community. Circulation. (2011) 123:2344–52. 10.1161/CIRCULATIONAHA.110.983536 21576654

[5] Levine GN Bates ER Blankenship JC Bailey SR Bittl JA Cercek B. 2011 ACCF/AHA/SCAI guideline for percutaneous coronary intervention: a report of the American College of Cardiology Foundation/American Heart Association Task Force on Practice guidelines and the society for cardiovascular angiography and interventions. Circulation. (2011) 124:e574–651. 10.1002/ccd.23390 22064601

[6] Thomas RJ King M Lui K Oldridge N Pi A IL Spertus J. AACVPR/ACC/AHA 2007 performance measures on cardiac rehabilitation for referral to and delivery of cardiac rehabilitation/secondary prevention services. J Am Coll Cardiol. (2007) 50:1400–33. 10.1016/j.jacc.2007.04.033 17903645

[7] Aragam KG Dai D Neely ML Bhatt DL Roe MT Rumsfeld JS. Gaps in referral to cardiac rehabilitation of patients undergoing percutaneous coronary intervention in the United States. J Am Coll Cardiol. (2015) 65:2079–88. 10.1016/j.jacc.2015.02.063 25975470

[8] Centers for Disease Control and Prevention (CDC). Receipt of outpatient cardiac rehabilitation among heart attack survivors–United States, 2005. CDC. (2008) 57:89–94. 18235423

[9] Dalal HM Zawada A Jolly K Moxham T Taylor RS. Home based versus centre based cardiac rehabilitation: cochrane systematic review and meta-analysis. BMJ. (2010) 340:b5631. 10.1136/bmj.b5631 20085991 PMC2808470

[10] Zwisler AD Norton RJ Dean SG Dalal H Taylor RS. Home-based cardiac rehabilitation for people with heart failure: a systematic review and meta-analysis. Int J Cardiol. (2016) 221:963–69. 10.1016/j.ijcard.2016.06.207 27441476

[11] Anderson L Sharp GA Norton RJ Dalal H Dean SG Jolly K. Home-based versus centre-based cardiac rehabilitation. Cochrane Database Syst Rev. (2017) 6:D7130. 10.1002/14651858.CD007130.pub4 28665511 PMC6481471

[12] Thomas RJ Beatty AL Beckie TM Brewer LC Brown TM Forman DE. Home-based cardiac rehabilitation: a scientific statement from the american association of cardiovascular and pulmonary rehabilitation, the American Heart Association, and the American College of Cardiology. J Am Coll Cardiol. (2019) 74:133–53. 10.1097/HCR.0000000000000447 31097258 PMC7341112

[13] Doherty PJ. The National Audit of Cardiac Rehabilitation: Annual Statistical Report 2017. London: British Heart Foundation (2017).

[14] Worringham C Rojek A Stewart I. Development and feasibility of a smartphone, ECG and GPS based system for remotely monitoring exercise in cardiac rehabilitation. PLoS ONE. (2011) 6:e14669. 10.1371/journal.pone.0014669 21347403 PMC3036581

[15] Korzeniowskakubacka I Dobraszkiewiczwasilewska B Bilińska M Rydzewska E Piotrowicz R. Two models of early cardiac rehabilitation in male patients after myocardial infarction with preserved left ventricular function: comparison of standard out-patient versus hybrid training programmes. Kardiol Pol. (2011) 69:220–26. 21432787

[16] Blasco A Carmona M Fernández-Lozano I Salvador CH Alonso-Pulpón L. Evaluation of a telemedicine service for the secondary prevention of coronary artery disease. J Cardiopulm Rehabil. (2011) 32:25–31. 10.1097/HCR.0b013e3182343aa7 22113368

[17] Rawstorn JC Gant N Direito A Beckmann C Maddison R. Telehealth exercise-based cardiac rehabilitation: a systematic review and meta-analysis. Heart. (2016) 102:1183–92. 10.1136/heartjnl-2015-308966 26936337

[18] Varnfield M Karunanithi M Lee CK Honeyman E Arnold D Hang D. Smartphone-based home care model improved use of cardiac rehabilitation in postmyocardial infarction patients: results from a randomised controlled trial. Heart. (2014) 100:1770–79. 10.1136/heartjnl-2014-305783 24973083

[19] Dorje T Zhao G Tso K Wang J Chen Y Tsokey L. Smartphone and social media-based cardiac rehabilitation and secondary prevention in China (SMART-CR/SP): a parallel-group, single-blind, randomised controlled trial. Lancet Dig Health. (2019) 1:e363–74. 10.1016/S2589-7500(19)30151-7 33323210

[20] Neubeck L Lowres N Benjamin EJ Freedman SB Coorey G Redfern J. The mobile revolution–using smartphone apps to prevent cardiovascular disease. Nat Rev Cardiol. (2015) 12:350–60. 10.1038/nrcardio.2015.34 25801714

[21] Yudi MB Clark DJ Tsang D Jelinek M Kalten K Joshi S. SMARTphone-based, early cardiac REHABilitation in patients with acute coronary syndromes [SMART-REHAB Trial]: a randomized controlled trial protocol. BMC Cardiovasc Disor. (2016) 16:170. 10.1186/s12872-016-0356-6 27596569 PMC5011930

[22] Jolly K Lip G Taylor RS Raftery J Mant J Lane D. The Birmingham rehabilitation uptake maximisation study (BRUM): a randomised controlled trial comparing home-based with centre-based cardiac rehabilitation. Heart. (2009) 95:36–42. 10.1136/hrt.2007.127209 18332063

[23] Spitzer RL Kroenke K Williams J Löwe B. A brief measure for assessing generalized anxiety disorder: the GAD-7. Arch Intern Med. (2006) 166:1092–97. 10.1001/archinte.166.10.1092 16717171

[24] Kroenke K Spitzer RL Williams J. The PHQ-9 validity of a brief depression severity measure. J Gen Intern Med. (2001) 16:606–13. 10.1046/j.1525-1497.2001.016009606.x 11556941 PMC1495268

[25] Spertus JA Winder JA Dewhurst TA Deyo RA Prodzinski J Mcdonnell M. Development and evaluation of the Seattle Angina Questionnaire: a new functional status measure for coronary artery disease. J Am Coll Cardiol. (1995) 25:333–41. 10.1016/0735-1097(94)00397-9 7829785

[26] O'Gara PT Kushner FG Ascheim DD Casey DEJ Chung MK de Lemos JA. 2013 ACCF/AHA guideline for the management of ST-elevation myocardial infarction: a report of the American College of Cardiology Foundation/American Heart Association Task Force on Practice Guidelines. Circulation. (2013) 127:e362–425. 10.1161/CIR.0b013e3182742c84 23247304

[27] Pescatello L Arena R Riebe D P T. ACSM's Guidelines for Exercise Testing and Prescription. 9th ed. Wolters Kluwer/Lippincott. Philadelphia: Williams & Wilkins (2014).

[28] Borg GA. Psychophysical bases of perceived exertion. Med Sci Sport Exer. (1982) 14:377–81. 10.1249/00005768-198205000-00012 7154893

[29] Ge C Ma J Xu Y Shi YJ Zhao CH Gao L. Predictors of adherence to home-based cardiac rehabilitation program among coronary artery disease outpatients in China. J Geriatr Cardiol. (2019) 16:749–55. 10.11909/j.issn.1671-5411.2019.10.003 31700514 PMC6828607

[30] Austin PC. An introduction to propensity score methods for reducing the effects of confounding in observational studies. Multivariate Behav Res. (2011) 46:399–424. 10.1080/00273171.2011.568786 21818162 PMC3144483

[31] Rubin DB. Multiple Imputation for Nonresponse in Surveys. Vol. 18. New York: John Wiley & Sons (2004).

[32] Soga Y Yokoi H Ando K Shirai S Sakai K Kondo K. Safety of early exercise training after elective coronary stenting in patients with stable coronary artery disease. Eur J Cardiovasc Prev Rehabil. (2010) 17:230–34. 10.1097/HJR.0b013e3283359c4e 20051870

[33] Nakamura H Arakawa K Itakura H Kitabatake A Goto Y Toyota T. Primary prevention of cardiovascular disease with pravastatin in Japan (MEGA Study): a prospective randomised controlled trial. Lancet. (2006) 368:1155–63. 10.1016/S0140-6736(06)69472-5 17011942

[34] Ettehad D Emdin CA Kiran A Anderson SG Callender T Emberson J. Blood pressure lowering for prevention of cardiovascular disease and death: a systematic review and meta-analysis. Lancet. (2016) 387:957–67. 10.1016/S0140-6736(15)01225-8 26724178

[35] Aragam KG Moscucci M Smith DE Riba AL Gurm HS. Trends and disparities in referral to cardiac rehabilitation after percutaneous coronary intervention. Am Heart J. (2011) 161:544–51. 10.1016/j.ahj.2010.11.016 21392610

[36] Brown TM Hernandez AF Bittner V Cannon CP Ellrodt G Liang L. Predictors of cardiac rehabilitation referral in coronary artery disease patients: findings from the American Heart Association's get with the guidelines program. J Am Coll Cardiol. (2009) 54:515–21. 10.1016/j.jacc.2009.02.080 19643312 PMC2760436

