# Supplementary material for: Chinese Home-Based Cardiac Rehabilitation Model Delivered by Smartphone Interaction Improves Clinical Outcomes in Patients With Coronary Heart Disease
Source: Front Cardiovasc Med. 2021 Oct 5;8:731557. doi: 10.3389/fcvm.2021.731557 (PMC8523852; doi:10.3389/fcvm.2021.731557)
Supplement: Supplementary file 1 [file Data_Sheet_1.PDF]

Supplemental Material

Methods:

CPET Examining Processes

A symptom-limited CPET was performed on an electronically operated cycle ergometry (Schiller cs-200, the Switzerland) following an individual ramp protocol: 3 min of rest, 3 min of unloaded cycling, starting at 20W, followed by an increasing work rate of 5 to 25 W/min at each time to maximal tolerance. Work rate was increased until it reaching maximal tolerated work rate in 6 to 10 min of exercise. The pedaling was kept at 55–65 revolutions/min. The test stopped when patient requested to stop or any acute cardiac events such as suspicion of myocardial infarction, moderate to severe angina, hypotension, signs of poor perfusion, severe shortness of breath and serious arrhythmias occurred, or when patient started to show following signs: electrocardiographic changes, worsening chest pain, severe fatigue, dyspnea, significant hypertension and less serious arrhythmias. The test was also terminated when patient was not able to continue pedaling at 50 rpm or when the Borg score was higher than 18.

Results:

Table S1. Baseline characteristics for matched cohort (1:1 matching between HBCR and control)

	**Control (n=135)**	**HBCR (n=135)**	***P* value**
**Age (years)**	57.3 ± 9.0	57.2 ± 9.1	0.95
**Sex, No.**			
**Male participant (%)**	120 (88.9%)	120 (88.9%)	1.0
**Manual workers (%)**	43 (31.8%)	42 (31.1%)	0.91
**Length of follow-up**	28.9 ± 8.5	30.8± 7.3	0.26
**BMI (kg/m**(Thompson, Arena, Riebe, & Pescatello,2013)^2**)**	26.3 ± 2.5	26.4 ± 2.7	0.90
**WHR**	0.93 ± 0.05	0.93 ± 0.05	0.52

**Exercise History (%)**	80 (59.2%)	85 (62.9%)	0.08
**Smoking History (%)**	120 (88.9%)	119 (88.1%)	0.92
**Systolic Blood Pressure (mmHg)**	129.7±13.4	129.3±13.5	0.98
**Systolic Blood Pressure Target-reached (%)**	90 (66.7)	89 (65.9)	0.93
**Diastolic Blood Pressure (mmHg)**	80.4±10.1	80.8±10.1	0.97
**Diastolic Blood Pressure Target-reached (%)**	69 (51.1)	72 (53.3)	0.48
**Labs**			
**LDL (mmol/L)**	1.92±0.69	1.94±0.67	0.81
**LDL Target-reached (%)**	38 (28.1%)	40 (29.6%)	0.67
**Total Cholesterol (mmol/L)**	3.44±0.89	3.42±0.87	0.72
**Total Triglyceride (mmol/L)**	1.46±0.99	1.44±0.71	0.57
**Uric Acid (µmol/L)**	350.1±80.0	352.7±78.1	0.64
**Homocysteine (µmol/L)**	15.9±6.1	14.4±5.6	0.31
**Comorbidities**			
**Myocardial Infarction History (%)**	58 (42.9%)	60 (44.4%)	0.59
**Hypertension (%)**	81 (60.0%)	81 (60.0%)	1.0
**Hyperlipidemia (%)**	108(80.0%)	104 (77.0%)	0.22
**Diabetes Mellitus (%)**	25 (18.5%)	25 (18.5%)	1.0
**Echocardiography**			
**LVEF (%)**	57.0 ± 7.0	57.5 ± 7.5	0.59
**Regional Wall Motion Abnormality or Ventricular Aneurysm (%)**	15(11.1%)	17(12.6%)	0.691
**LVIDd (mm)**	46.4 ± 4.6	46.5 ± 4.8	0.861
**IVS (mm)**	10.8 ± 1.1	10.8 ± 1.3	0.974
**Percutaneous coronary intervention**			
**Number of Stents / patients**	2.2 ± 1.2	2.5 ± 1.6	0.071
**Untreated Stenosis (%)**	87 (52.7%)	100 (58.8%)	0.273

**Medication**			
**Anti-platelet (%)**	159 (96.4)	168 (98.8)	0.169
**Statins (%)**	133 (80.6)	138 (81.2)	0.502
**β-blocker (%)**	95(57.6)	104(60.6)	0.581
**ACEI/ARB (%)**	34(20.6)	40 (23.5)	0.513
**Nitrates (%)**	61(37.0)	64(37.6)	0.911
**Diltiazem (%)**	29(17.6)	33 (19.4)	0.676
**Trimetazidine (%)**	57 (34.5)	62(36.5)	0.733
**CPET**			
**METS**	5.60±1.31	5.27±1.32	0.070
**Peak Oxygen Pulse (ml O_2/beat)**	12.04±3.64	11.38±4.47	0.142
**VO_2 AT (ml.kg-1.min-1)**	15.08±4.09	14.13±4.71	0.050*
**VE/VCO_2**	25.07±4.81	25.92±4.26	0.086
**$\triangle VO_2/\triangle WR$ (ml.min^{-1}.W^{-1})**	12.05±3.13	11.39±2.69	0.039*
**Psychological Stress**			
**GAD-7**	3.62±4.60	2.87±3.63	0.107
**PHQ-9**	4.78±4.18	4.16±3.16	0.137
**Symptoms**			
**SAQ-PL**	68.15±13.95	66.08±15.41	0.279
**SAQ-AS**	64.53±28.39	65.31±30.31	0.837
**SAQ-AF**	88.84±18.77	88.78±16.82	0.977
**SAQ-TS**	78.44±14.92	80.06±13.41	0.377
**SAQ-DP**	61.85±22.25	59.43±19.71	0.371

Table S2. Missing rate of primary and second outcomes

	**Control (n=165)**	**HBCR (n=170)**
**PRIMARY OUTCOME**		

**MACE**		
**Incidence of Myocardial Infarction**	0%	0%
**Unscheduled Revascularization**	0%	0%
**Stroke**	0%	0%
**Cardiac death**	0%	0%
**SECOND OUTCOMES**		
**Unscheduled Hospitalization because of Worsened Angina**	0%	0%
**CPET**	1.2%	0.6%
**Risk factors control**		
**SBP**	0%	0%
**LDL**	47.3%	43.5%
**TC**	51.5%	47.1%
**TG**	52.7%	47.1%
**UA**	53.9%	48.2%
**HcY**	77%	73.5%
**Survey**		
**GAD-7**	9.7%	5.9%
**PHQ-9**	9.7%	5.3%
**SAQ**	41.8%	28.2%

BP: Blood Pressure; LDL: Low-Density Lipoprotein cholesterol; TC: Total cholesterol; TG, Total Triglyceride;

UA: Uric acid; Hcy, Homocysteine; GAD-7: Generalized Anxiety Disorder-7; PHQ-9: Generalized Anxiety Disorder-7; SAQ: Seattle Angina Questionnaire;

Table S3. Summary statistics of Home-Based Cardiac Rehabilitation program on primary and second outcomes

29

	Control (n=165)	HBCR (n=170)	$P^{\dagger}$
**PRIMARY OUTCOMES**			
**MACE**	14 (8.5)	4(2.4)	0.015*
**Incidence of Myocardial Infarction**	3 (1.8)	0 (0.0)	0.118
**Unscheduled Revascularization**	14 (8.5)	4 (2.4)	0.015*
**Stroke**	0 (0.0)	0 (0.0)	-
**Cardiac death**	1 (0.6)	0 (0.0)	0.493
**SECOND OUTCOMES**			
**Unscheduled Hospitalization because of Worsened Angina**	36 (21.8)	17 (10.0)	0.004*
**CPET**			
**METS**	5.57±1.38	6.08±1.43	0.001*
**Peak Oxygen Pulse (ml O2/beat)**	12.12±3.61	12.59±3.25	0.214
**VO2 AT (ml.kg-1.min-1)**	14.19±4.03	15.75±4.65	0.001*
**VE/VCO2**	25.32±3.75	24.90±3.88	0.316
**△VO2/△WR (ml.min-1.W-1)**	11.50±2.83	11.81±2.64	0.303
**Risk factors control**			
**SBP (mmHg)**	129.1±13.64	125.2±13.94	0.010*
**Target- reached Ratio of SBP**	79 (47.9)	119 (70)	0.000*
**DBP (mmHg)**	80.3±10.35	78.4±11.14	0.118
**Target- reached Ratio of DBP**	84 (50.9)	103 (60.6)	0.079
**LDL (mmol/L)**	2.19±0.80	1.66±0.44	0.000*
**Target- reached Ratio of LDL**	58 (35.2)	122 (71.8)	0.000*
**Other Labs**			
**TC (mmol/L)**	3.56±1.15	3.22±0.74	0.021*
**TG (mmol/L)**	1.57±1.09	1.37±0.79	0.164

**UA (mmol/L)**	341.62±85.39	344.18±75.94	0.841

P[†] is the p-value for raw data (without imputation);
METS indicates Metabolism Equivalents; VO_2 AT, oxygen consumption at Anaerobic Threshold; VE, pulmonary ventilation; VCO_2, carbon dioxide production; VE/VCO_2, minute ventilation/carbon; dioxide production relationship; △VO_2/△WR, VO_2/work rate relationship. SBP indicates Systolic Blood Pressure; DBP, Diastolic Blood Pressure; LDL, Low-Density Lipoprotein cholesterol; TC, Total Cholesterol;
TG, Total Triglyceride; UA, Uric Acid.
Data are shown as mean ± standard deviation (SD).
Time effects of exercise(±) on the second endpoints were analyzed using paired t-test.*P-value <0.05 was considered to be of statistical significance.

Table S4. Multivariate Cox regression analysis of the correlation between Home-Based Cardiac Rehabilitation program and incidence of clinical events

	**HR (95%CI)**	***P* value**
**crude**	0.274 (0.090, 0.833)	0.022
**Model 1**	0.274 (0.090, 0.833)	0.022
**Model 2**	0.274 (0.090, 0.833)	0.022
**Model 3**	0.273 (0.090, 0.828)	0.022

Model 1: after adjustment for age and gender.
Model 2: after further adjustment for history of previous myocardial infarction, history of diabetes mellitus, number of stents implanted, whether has residual stenosis or not.
Model 3: after further adjustment for BMI, WHR, history of Hypertension , history of smoking, history of exercise, maximal METs, oxygen uptake at AT, VE/VCO2, △VO2/△WR, use of at least one antiplatelet drug, use of statins, use of trimetazidine, use ofβ-blockade, Whether received emergency PCI or not, systolic blood pressure value and diastolic blood pressure value at the baseline level.
BMI indicates Body Mass Index; WHR, Waist Hip Rate; METS indicates Metabolism Equivalents; AT, Anaerobic Threshold; VE/VCO2, minute ventilation/carbon dioxide production relationship; △VO2/△WR, VO2/work rate relationship; PCI, percutaneous coronary intervenetion.

Table S5. Effects of Home-Based Cardiac Rehabilitation program on psychological state, cardiac symptoms and biochemical Metric at the last follow-up

	Control † (n=165) Mean (95%CI)	HBCR † (n=170) Mean (95%CI)	*P*††
**Psychological Stress**			
**GAD-7**	3.55±4.08	2.63±3.40	0.029*
**PHQ-9**	4.96±3.83	4.03±3.08	0.018*
**Symptoms**			
**SAQ-PL**	67.36±14.72	71.90±10.57	0.003*
**SAQ-AS**	60.53±30.80	69.48±24.56	0.008*
**SAQ-AF**	90.60±13.80	93.10±13.31	0.125
**SAQ-TS**	76.43±16.91	82.27±14.21	0.002*
**SAQ-DP**	65.14±20.12	67.98±19.91	0.238

† Mean values were summarized based on imputed data. ***P*††** is the p-value after imputation to test whether the mean is the same between
HBCR and control groups
GAD-7 indicates Generalized Anxiety Disorder-7; PHQ-9, Generalized Anxiety Disorder-7; SAQ,
Seattle Angina Questionnaire; PL, Physical Limitation; AS, Anginal Stability; AF, Anginal Frequency;
TS, Treatment Satisfaction; DP, Disease Perception.
**P*-value <0.05 was considered to be of statistical significance.

Figure S1

KM curve by the raw data in control and HBCR groups

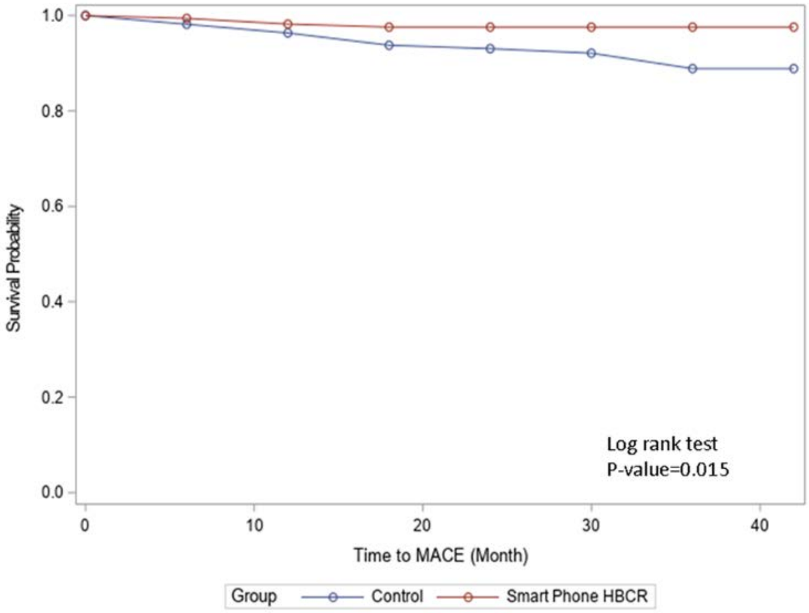
1.0
0.8
0.6
0.4
0.2
0.0
Survival Probability
0
10
20
30
40
Time to MACE (Month)
Log rank test
P-value=0.015
Group
Control
Smart Phone HBCR
